# A computationally efficient hybrid framework combining deep feature extraction and gradient boosting for early diagnosis of Olive leaf diseases

**DOI:** 10.1038/s41598-025-31918-x

**Published:** 2025-12-11

**Authors:** Uğur Şevik, Fatih Serdar Aydemir

**Affiliations:** 1 Department of Computer Science, Faculty of Science, Karadeniz Technical University, Kanuni Campus, 61080 Ortahisar, Trabzon, Türkiye; 2 Retina R&D Software and Engineering Services Ltd, Trabzon Teknokent, Trabzon, Türkiye

**Keywords:** Olive leaf diseases, Transfer learning, Machine learning, Hybrid classification model, Plant disease detection, Computational biology and bioinformatics, Mathematics and computing, Plant sciences

## Abstract

Olive production holds a significant position in the global agricultural trade. In addition to being influenced by seasonal climatic conditions, agricultural diseases are another factor that affects olive yield. Peacock spot disease and olive bud mites are the primary agricultural diseases affecting olive production. These two diseases cause specific lesions in the leaves of olive trees. It has been observed that artificial intelligence approaches such as deep learning and machine learning are used for early detection of such adverse conditions. However, the need for high computational processing in the classification and detection processes of deep learning models limits the accessibility of these algorithms to all businesses. To address this challenge, this study proposes a hybrid framework that combines the robust feature extraction capabilities of deep learning models with the computational efficiency of machine-learning classifiers. Specifically, this study analyzes the performance of this combined approach and compares the results with those of existing deep learning studies in the literature. As feature extraction deep learning models, MobileNetV2, DenseNet121, EfficientNetV2B0, and ConvNext Tiny were selected, while AdaBoost, XGBoost, LightGBM, CatBoost, and Gradient Boosting algorithms from the Boosting family were included as classifiers. In the model training, a dataset consisting of 3,400 images of olive leaves belonging to three classes—healthy, olive_peack_spot, and aculus_olearius—was used. The experimental results showed that the DenseNet121 + XGBoost combination achieved a baseline accuracy of 92%. Following a data augmentation phase to enrich the training data, the model performance was significantly enhanced, reaching a final accuracy of 94% and a macro average F1-Score of 94%. The Wilcoxon Signed-Rank test revealed that the DenseNet121 + XGBoost combination statistically outperformed the second-best model (*p* < 0.05). This performance is attributed to the dense connectivity of DenseNet, which promotes effective feature reuse and improves gradient flow. Furthermore, the study demonstrates that a higher number of parameters does not always guarantee better performance; rather, architectural efficiency plays a crucial role in avoiding overfitting and ensuring model robustness, as evidenced by DenseNet121 outperforming the larger ConvNeXtTiny model.

## Introduction

Olives are one of the primary agricultural products exported by countries in the Mediterranean Basin. Globally, the majority of olive groves and olive production are concentrated in this region. Unlike many other agricultural products, olive production is highly geographically concentrated. Data published by the Food and Agriculture Organization (FAO) and International Olive Council (IOC) indicate that over 90% of global olive production occurs in countries located in the Mediterranean Basin^1^. Spain is the world leader in terms of both the size of the cultivated area and production volume. Following Spain, Italy, Greece, Tunisia, and Turkey are the leading olive-producing countries^2^. Turkey ranks among the top three countries in the world for table olive production and among the top five for olive oil production. Currently, the number of olive trees in the country exceeds 190 million^3^.

**Fig. 1:**
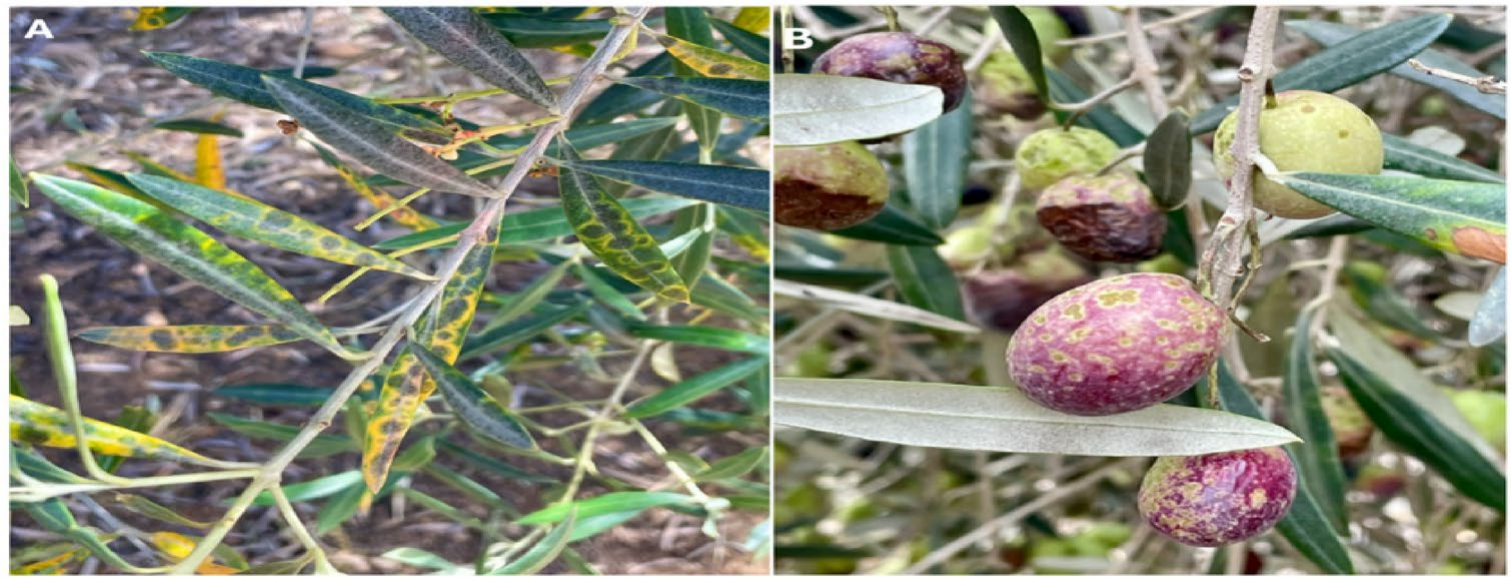
Ring Spot Disease observed on the leaves and fruit of the olive tree^8^.

The yield obtained from olive production largely depends on the climatic conditions during the production season and the severity of agricultural diseases affecting olive trees. Among these, Olive Leaf Spot (Spilocaea oleagina) and Olive Bud Mite (Aculus olearius) are the most prominent. Olive Leaf Spot, which commonly occurs in mild and rainy climates, is a plant disease that creates circular spots on the leaves. Because of this feature, it is also popularly known as “Olive Peacock Spot.” An example of this disease is illustrated in Fig. 1. By covering the surface of the leaves and narrowing the photosynthetic area, this disease disrupts the physiological balance of olive trees and indirectly reduces their yield. This physiological disturbance can cause severe yield losses in subsequent production years^4^. In contrast, the Olive Bud Mite directly damages the buds, flowers, and young fruits of the tree, causing bud blackening, flower drop, and fruit deformities^5^. Studies indicate that if left untreated, these diseases can lead to significant yield losses, estimated to reach 20%–30% in severe cases^3,6^. This poses a critical economic threat, particularly to the Mediterranean basin, where mild and rainy climatic conditions favor pathogen spread and where over 90% of global olive production is concentrated^1,7^.

Because plant diseases directly affect the quality and yield of agricultural products, early detection of these diseases is crucial. Currently, artificial intelligence (AI)-supported decision-making systems are effectively used to address such issues. Alshammari et al. (2023) proposed a hybrid model combining the Whale Optimization Algorithm (WOA) with Artificial Neural Networks, achieving an accuracy rate of 98.9% on a dataset consisting of 950 healthy, 890 olive bud mite (Aculus olearius) infected, and 1460 peacock spot disease (Cycloconium oleaginum)-infected olive leaves^9^. Raouhi and his team conducted an experimental study by hybridly integrating various Convolutional Neural Network (CNN) algorithms with optimization methods on a dataset containing 5571 images of six different olive leaf diseases. By combining the MobileNet and RMSProp algorithms, an accuracy of 98.48% was achieved^10^. Sinha et al. compared histogram equalization and k-means clustering techniques to isolate disease regions and found that the k-means method provided higher accuracy^11^. Dikici et al. trained deep learning models, including AlexNet, SqueezeNet, ShuffleNet, and GoogleNet, to classify olive leaf diseases and compared these models. The best result was obtained using the ShuffleNet al.gorithm, with an accuracy of 98.52%^12^. Sarantakos and his team comparatively examined the CNN, Vision Transformer (ViT), and Amazon Web Services (AWS) Rekognition systems, and found that the highest accuracy, 99.6%, was achieved with AWS Rekognition^13^. In another study, Ksibi et al. developed a model called MobiRes, which is a combination of MobileNet and ResNet, by collecting 5400 images with an unmanned aerial vehicle from an olive grove in Saudi Arabia. The model produced successful results with an accuracy of 97.08%^14^. In another study by Alshammari, a hybrid approach combining the VGG16 and Vision Transformer algorithms was proposed, achieving 96% accuracy in a multi-class classification task^15^. In a study conducted by Huaquipaco et al., an architecture called SSl-XceVnet, which combined the Xception and VGG16 algorithms, was proposed for detecting peacock spots, and an accuracy rate of 95.22% was achieved^16^. Uğuz and Uysal compared their self-developed CNN-based model with VGG16 and VGG19 models. In tests performed on a dataset of 3400 images, they achieved 88% accuracy with the normal dataset and 95% with the augmented dataset, outperforming other competing models^17^. The FLVAEGWO-CNN model proposed by Majikumna et al. presented a hybrid structure combining a variational autoencoder (VAE), grey wolf optimization (GWO), and convolutional neural networks. The experiments achieved an accuracy of 99.2%^18^. Dammak et al. developed a two-stage model in which the first stage detected leaves and the second stage classified the disease type. The proposed system achieved high success rates in classification tasks^19^.

Recent advancements in precision agriculture have highlighted the efficacy of ensemble learning and intelligent decision-making systems for plant disease diagnosis. Studies have demonstrated that ensemble strategies, which aggregate predictions from multiple deep learning architectures, significantly enhance diagnostic accuracy and robustness compared with single models^20–23^. Furthermore, the integration of Explainable AI (XAI) has gained traction, addressing the black-box nature of deep learning by providing interpretable insights into disease classification features^22^. Novel frameworks that combine deep feature extraction with optimization algorithms and hybrid classifiers are being developed to support real-time monitoring and automated decision-making in agricultural environments^21,24^. These studies collectively underscore the growing trend toward the development of computationally efficient and high-performance hybrid models for intelligent agricultural applications in the future.

In this study, a novel hybrid approach is proposed for the detection of olive leaf diseases by combining the automatic feature extraction capability of deep learning models, which offer high accuracy rates, with the low computational cost of machine learning algorithms. Owing to this hybrid structure, the high hardware requirements of deep learning are reduced while preserving its capacity to learn meaningful features from data. The developed approach aims to facilitate access to technology for farmers operating at different scales and provide cost-effective solutions.

During the testing phase of the proposed hybrid model, ConvNextTiny, DenseNet121, MobileNetV2, and EfficientNetV2B0 were used as feature-extracting deep learning models, whereas the AdaBoost, LightGBM, XGBoost, CatBoost, and GradientBoosting algorithms were utilized as classifiers. Each deep learning model was paired with each machine learning algorithm, creating 20 different combinations that were experimentally compared. The two most successful model combinations were also evaluated using statistical tests, which demonstrated that the high accuracy rates obtained were statistically significant.

The developed hybrid approach has the potential to detect common diseases in olive groves, such as Peacock Spot (Spilocaea oleagina) and Olive Bud Mite (Aculus olearius), before they spread. Early disease diagnosis helps limit environmental impacts by reducing pesticide use and supports the economic sustainability of small- and medium-sized producers. Moreover, minimizing product loss increases the yield.

In line with the successful results, the development of a user-friendly mobile application for small- and medium-sized producers is feasible. For large-scale olive producers, smart scanning systems integrated with unmanned aerial vehicles are recommended to enable the monitoring of larger areas. Thus, it aims to offer a scalable, flexible, and technologically accessible solution for producers of various sizes.

## Materials and methods

**Fig. 2:**
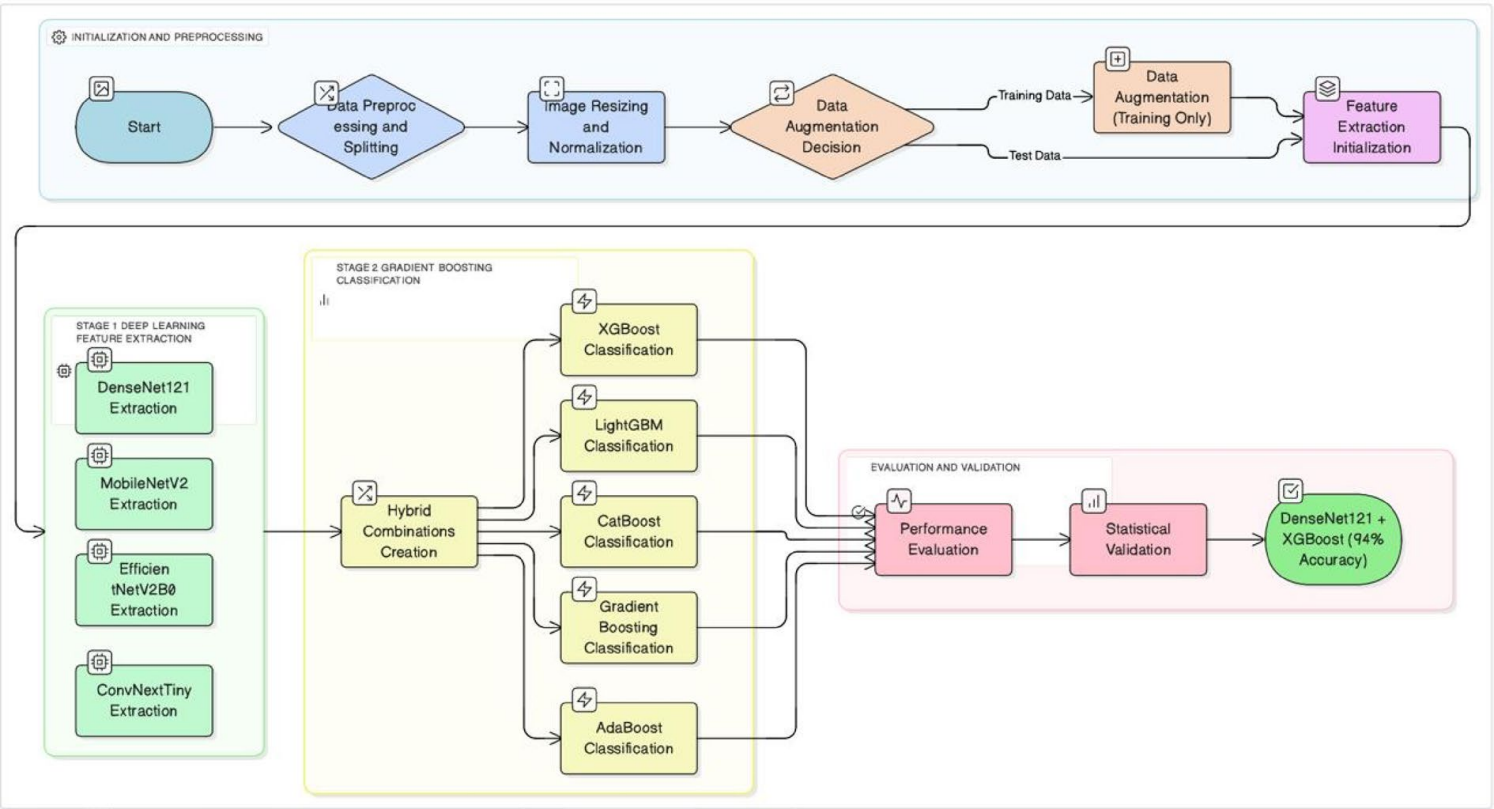
Comprehensive workflow of the proposed hybrid approach. The process is divided into two main stages: (1) Deep Learning-based feature extraction using pretrained CNN models and (2) classification using Gradient Boosting algorithms. The data augmentation step was applied exclusively to the training data to ensure an unbiased evaluation.

In this study, a hybrid classification approach that combines deep learning and machine learning algorithms was proposed for the detection of diseases observed in the leaves of olive plants, which hold a significant place in global agricultural trade. During the feature extraction phase, deep learning-based models ConvNextTiny, DenseNet121, MobileNetV2, and EfficientNetV2B0 were used. The feature matrices obtained using these models were evaluated in the classification process using methods from the boosting algorithm family, including AdaBoost, LightGBM, XGBoost, CatBoost and Gradient Boosting. The testing process of the proposed hybrid approach was conducted within the framework of a workflow diagram, in which the modeling steps were systematically followed. The overall flow and modeling steps related to this process are shown in Fig. 2.

### Data set

**Fig. 3:**
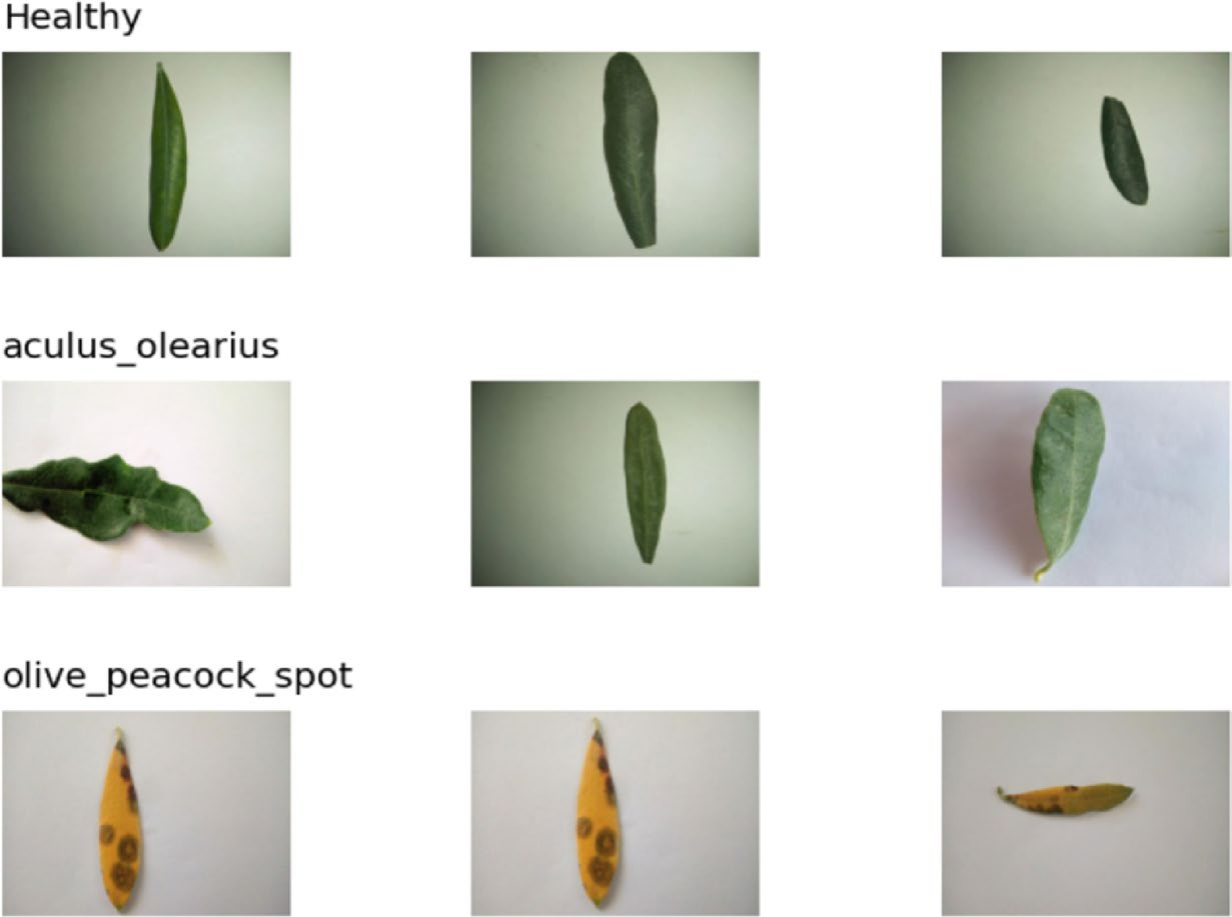
Sample images from each class in the dataset are shown.

The dataset used in this study was obtained from the open-access data science platform Kaggle. The images were based on real field data collected from an olive orchard in Denizli, Turkey^25^. The dataset consisted of 3,400 images belonging to three different classes. The classes were categorized as healthy leaves, olive peacock spots, and olive bud mites (Aculus olearius). The numbers of sample images for each class were 1,050, 1,460, and 890. Sample images from the dataset are presented separately for each class in Fig. 3, and detailed numerical information regarding the class distribution is provided in Table 1.

**Table 1:** Distribution of images belonging to each class in the dataset.

Class name	Number of images
Healthy	1050
Olive Peacock Spot	1460
Aculus Olearius	890
Total	3400

The images in the dataset are real-world field images captured under natural lighting conditions rather than controlled laboratory samples. As detailed in Table 1, there is a noticeable class imbalance (e.g., 1,460 images for Olive Peacock Spot vs. 890 for Aculus Olearius). To mitigate the potential bias arising from this imbalance before the data augmentation phase, we relied on the robust handling capabilities of the selected Gradient Boosting classifiers and prioritizing the macro average F1-Score over accuracy as the primary metric for performance evaluation.

#### Data preprocessing step

Before proceeding to the feature extraction phase using deep learning models, a standard preprocessing procedure was applied to all images in the dataset. In the first step, each image was resized according to the specific input requirements of the respective models, as listed in Table 2^26^. During this process, the LANCZOS interpolation filter was used to preserve the image quality and structural integrity by calculating the weighted average of the surrounding pixels^27^. Subsequently, normalization was applied by dividing the pixel values by 255 to scale them to the 0–1 range, ensuring gradient balance and training stability. Following these steps, the dataset consisting of 3,400 samples was split into two parts with an 80% training (2,720 images) and 20% testing (680 images) ratio, respectively. To further enhance the generalization capability of the models and prevent overfitting, data augmentation techniques were applied dynamically during the training phase using the Keras library. These transformations included random rotations of up to 20 °, width and height shifts of 10%, shear and zoom variations of up to 10%, and horizontal flipping, thereby increasing the dataset diversity.

**Table 2:** Number of parameters, number of layers, and input size of the models used.

Model name	Number of parameters	Number of layers	Input size
ConvNextTiny	28.6 Million	50	224 × 224
DenseNet121	8.1 Million	242	224 × 224
MobileNetV2	3.5 Million	105	150 × 150
EfficientNetV2B0	7.2 Million	55–60	224 × 224

### Proposed hybrid approach

The proposed hybrid approach is based on a two-stage model architecture that combines deep learning-based feature extraction with machine-learning-based classification processes. The main goal of this approach is to leverage the automatic feature extraction capability of Convolutional Neural Networks (CNNs) from visual data using transfer learning and to classify these features with machine learning algorithms whose accuracy has been scientifically validated. Thus, by eliminating the need for traditional feature engineering, a more efficient, generalizable, and low-cost solution was achieved. The proposed hybrid approach consists of two stages: feature extraction and classification, which are configured using deep learning-based models and machine-learning algorithms, respectively.

#### Feature extractor deep learning models

In the initial stage of the model architecture, a transfer learning approach was adopted to automatically extract meaningful and distinctive features from olive tree leaf images. This method allows CNN-based models that have been pretrained on large-scale datasets containing millions of images, such as ImageNet^28^, to be used not only for classification purposes but also for feature extraction. Thus, while avoiding the high computational cost that would arise from training a model from scratch, more stable and faster learning processes have been achieved by utilizing general-purpose visual representations of the models. Within this scope, four CNN models that stand out in the literature owing to their high accuracy rates and architectural diversity were chosen as feature extractors: MobileNetV2^29^, EfficientV2B0^30^, DenseNet121^31^, and ConvNeXtTiny^32^. The weights trained on ImageNet for these models were retained. Specifically, the top fully connected layers were removed, and feature vectors were extracted directly from the Global Average Pooling layer of each architecture. This ensured that the spatial dimensions collapsed into a meaningful 1D feature vector representing the entire image content. In this study, a frozen-layer training strategy was adopted. The convolutional base of each model was initialized with pre-trained ImageNet weights and maintained in a non-trainable (frozen) state throughout the process of training. This strategic decision is underpinned by both dataset constraints and the computational efficiency. Primarily, given the current dataset size of 3,400 images, subjecting deep architectures to full fine-tuning poses a significant risk of overfitting; thus, preserving pre-trained robust features enhances the model generalization capability. Additionally, freezing the model backbone eliminates the necessity for backpropagation across millions of parameters, thereby minimizing the computational load and aligning perfectly with the core objective of this study of providing an accessible, cost-effective solution. Thus, for each input image, high-dimensional feature vectors were obtained through the convolutional layers of the models. These features were then used as input data for the machine learning algorithms employed in the next step. The role of the selected models in this study and the reasons for their selection are summarized below.

##### MobileNetV2

**Fig. 4:**
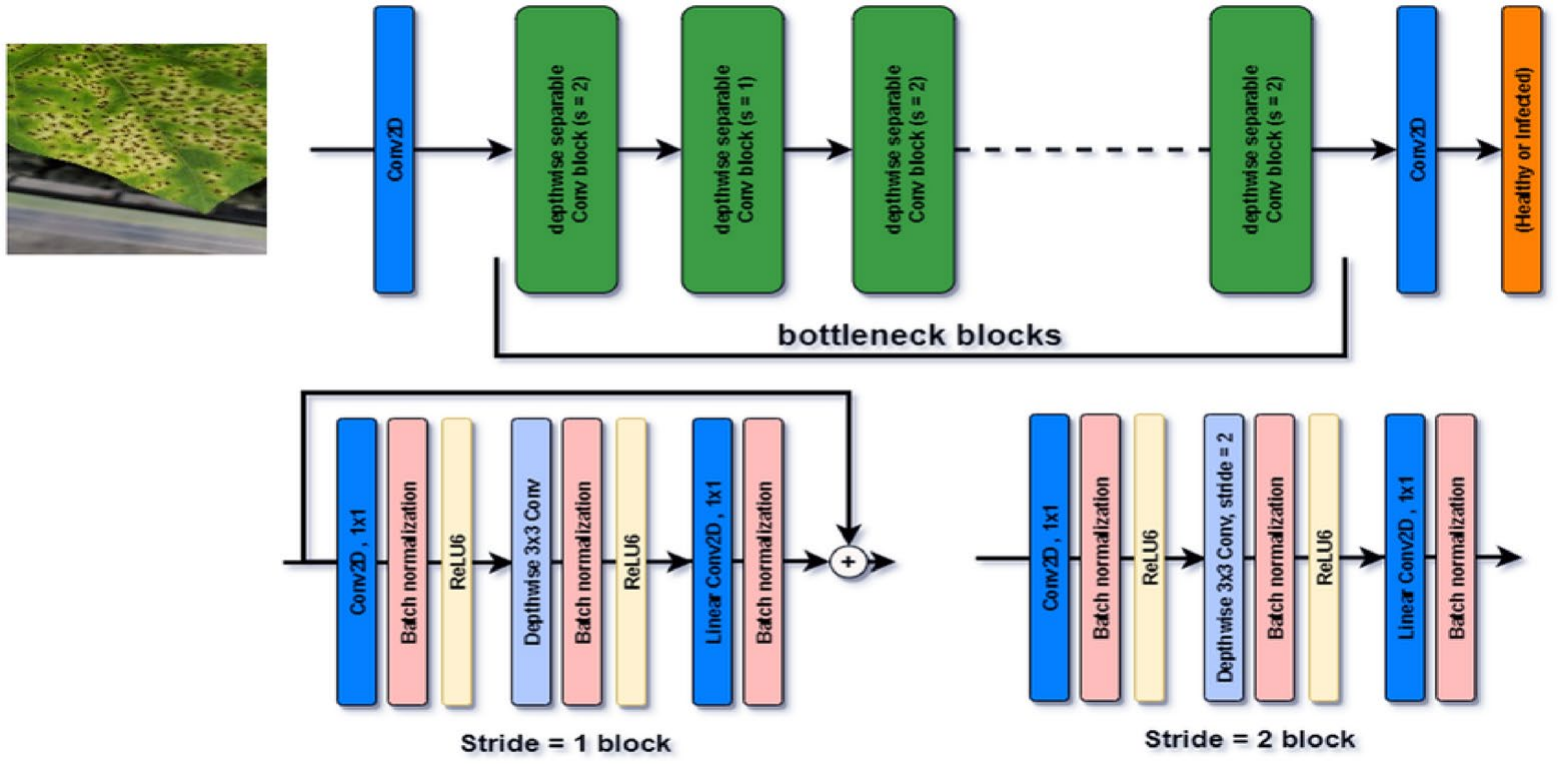
MobileNetV2 Architecture^33^.

The MobileNetV2 model was primarily included in this study to evaluate its computational efficiency and rapid feature extraction performance. The depth-wise separable convolutional blocks used in its architecture, as illustrated in Fig. 4, significantly reduce the number of parameters and computational cost of the model. This structural advantage makes the model an ideal candidate for testing its potential in scenarios such as real-time mobile agricultural applications, drone-based image processing, and embedded systems with limited hardware.

##### EfficientNetV2B0

**Fig. 5:**
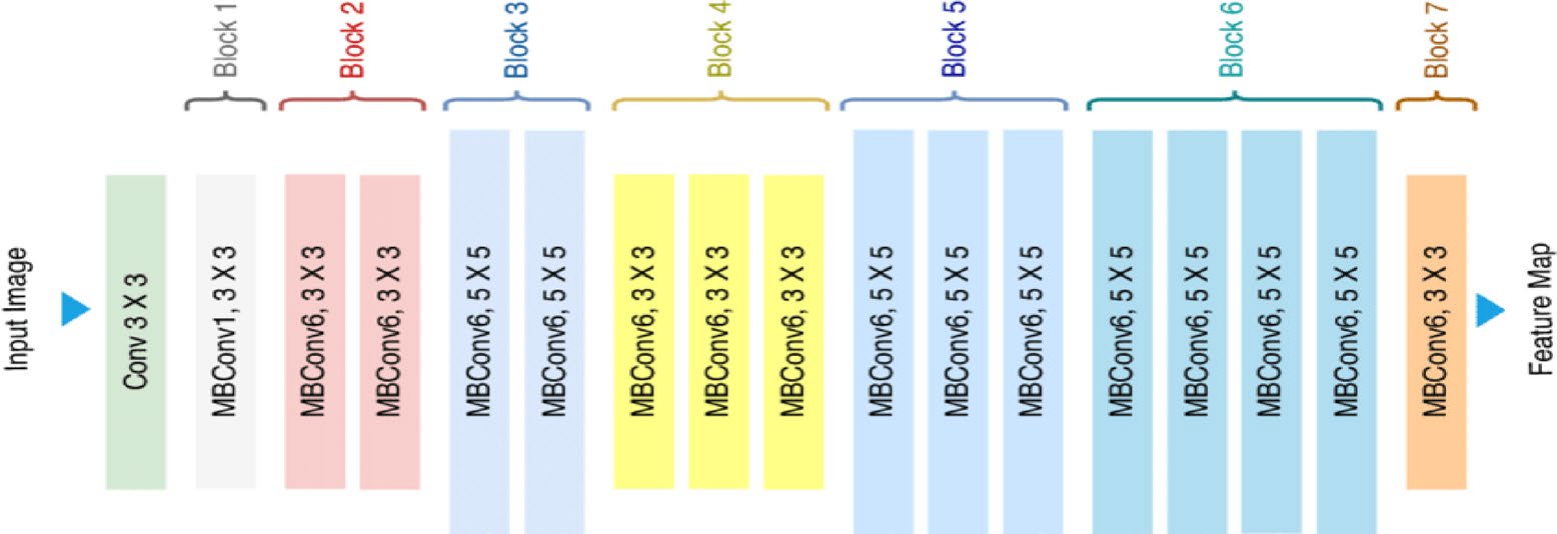
EfficientNetV2B0 Architecture^34^.

The EfficientV2B0 model was included because it represents a modern architecture that offers an optimal balance between model size, inference speed, and classification accuracy. The compound scaling principle, which forms the foundation of this model family, enables the network to achieve high performance and efficiency by systematically scaling key parameters, such as depth, width, and input resolution. This balanced structure, depicted in Fig. 5, makes the model a strong candidate, particularly for general-purpose and cloud-based agricultural analysis services that do not focus on a single metric. Therefore, EfficientNetV2B0 is considered an ideal architecture, especially for general-purpose use cases such as cloud-based agricultural decision support systems.

##### DenseNet121

**Fig. 6:**
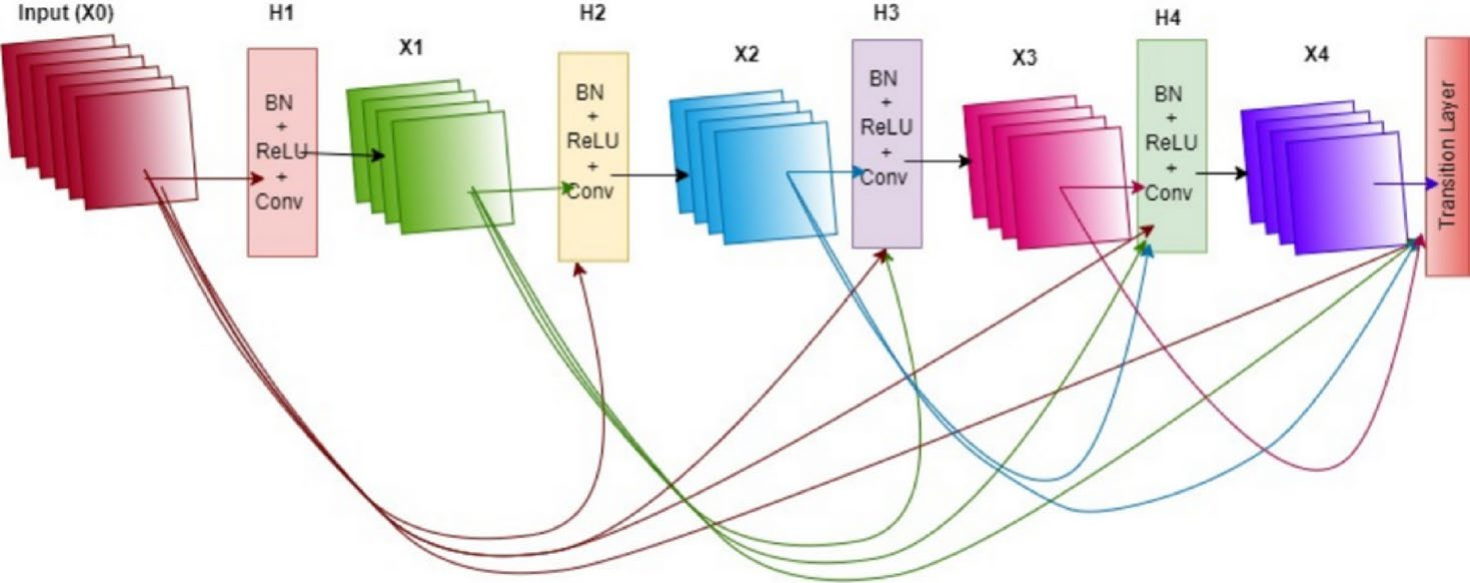
DenseNet121 architecture^35^.

The DenseNet121 model was included in this study to analyze the performance of an architecture with a high parameter efficiency. The distinguishing feature of this model is its densely connected structure, in which each layer is directly connected to all preceding layers. This architecture, shown in Fig. 6, encourages the effective reuse of features across layers while allowing gradients to flow through the network without vanishing, thus enhancing the learning process. As a result, DenseNet121 has the potential to offer a strong learning capacity with fewer parameters, making it a notable option, especially for systems with memory constraints but high accuracy requirements.

##### ConvNextTiny

**Fig. 7:**
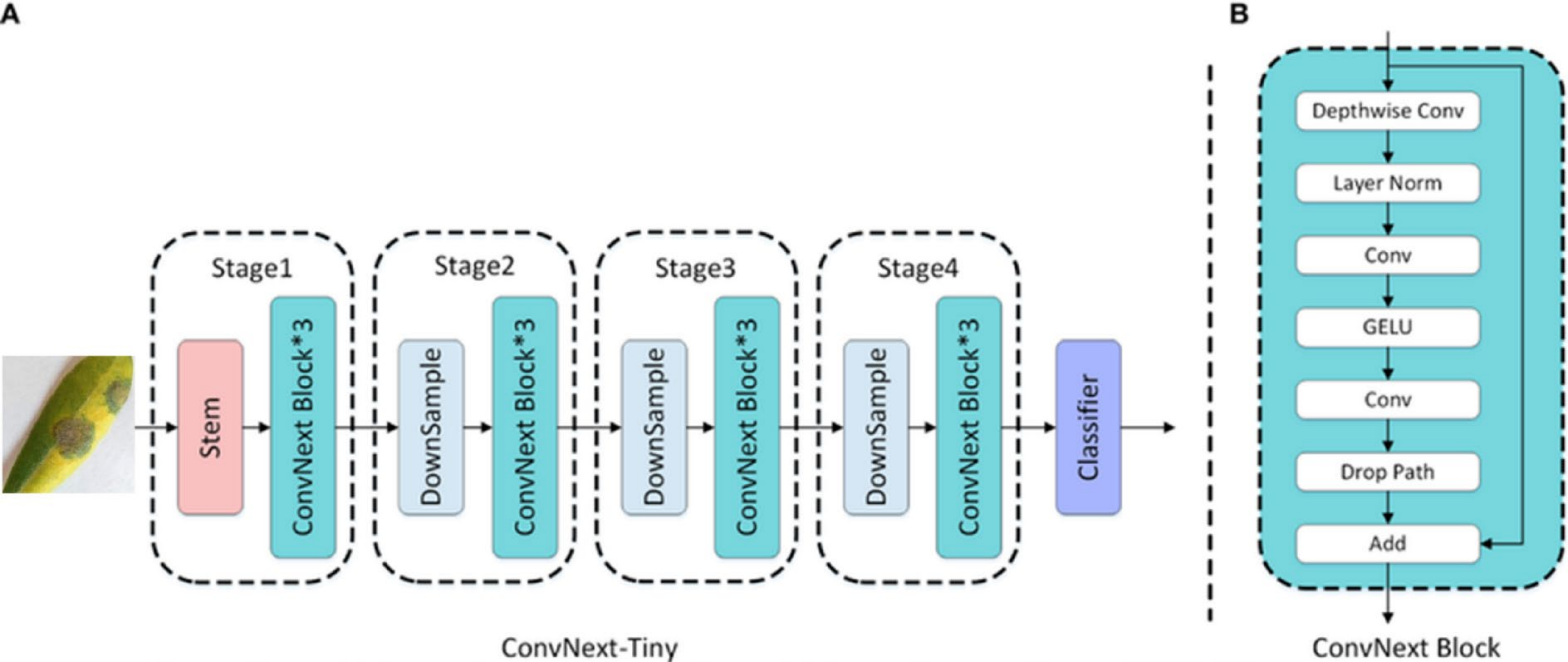
ConvNext-tiny architecture^36^.

The ConvNextTiny model is a modernized version of the traditional Convolutional Neural Network architecture inspired by transformer-based structures. In this model, 7 × 7 large kernels were used instead of the standard small kernels, allowing for a larger receptive field at each layer. Consequently, the model can learn broader contextual relationships within images. This modification, together with other modern components such as layer normalization, GELU activation, and depthwise convolution, forms the overall architecture, as shown in Fig. 7. Despite its compact structure, ConvNextTiny demonstrated high classification success, making it a suitable candidate for applications such as offline decision support systems, where accuracy is critical and inference speed is of secondary importance.

Following the feature extraction stage, each image was represented by a feature vector whose dimensions were determined by the specific architecture of the base model. The resulting vector dimensions were 1024 for DenseNet121, 1280 for MobileNetV2, 1280 for EfficientNetV2B0, and 768 for ConvNeXtTiny models. These feature vectors were then used as inputs for the classifier machine learning algorithms in the next stage.

#### Classifier machine learning models

In the second stage of the hybrid approach, five gradient boosting algorithms based on the ensemble learning principle AdaBoost^37^, CatBoost^38^, GradientBoosting^39^, LightGBM^40^, and XGBoost^41^, were selected to classify the numerical feature vectors obtained from the deep learning models. These algorithms have been prioritized over traditional methods, such as Support Vector Machines (SVM) and random forests, primarily because of their sequential learning architecture. Unlike SVM, which can become computationally intensive with high-dimensional feature vectors, and Random Forest, which relies on independent decision trees, boosting methods actively correct the errors of the predecessor models. This sequential error correction mechanism allows for superior optimization of the loss function, effectively mitigating both bias and variance in complex agricultural datasets. Furthermore, modern implementations such as XGBoost and LightGBM incorporate L1/L2 regularization, providing enhanced resistance to overfitting. Consequently, this comprehensive configuration aims to identify the feature extractor-classifier pairing that yields the highest classification performance. The reasons for selecting the preferred algorithms are as follows:

**Adaptive boosting** (AdaBoost) is a pioneer ensemble learning algorithm that enables the transformation of weak classifiers into strong classifiers by training them sequentially. In each iteration, the learning process of the model was guided by assigning more weight to the examples misclassified by the previous classifier. Owing to this dynamic weight-updating structure, the model achieves a high generalization capacity. In our study, AdaBoost was considered the primary reference model and served as a benchmark for evaluating the performance of other modern boosting methods. Owing to its simplicity, interpretability, and low requirements for parameter tuning, it was used as the initial benchmark in the modeling process.

**The gradient boosting** The (GB) algorithm aims to increase prediction accuracy through sequentially added weak classifiers that seek to minimize errors. Each new model focuses on improving the error terms produced by the previous model in the gradient direction. This gradient-based optimization mechanism is notable for its flexibility in guiding the learning process according to the loss function. In this respect, GB has a more powerful and flexible structure than AdaBoost. In our study, it was included as a comparative reference to classical boosting approaches, as it forms the basis for modern and faster variants, such as XGBoost and LightGBM.

**Extreme gradient boosting (XGBoost**) is an optimized version of the Gradient Boosting algorithm and is frequently used in industrial applications. Among its most notable features are support for parallel computation, sparse data, resilience against missing data, and integrated L1 and L2 regularization techniques to prevent overfitting. Consequently, both high classification accuracy and improved model generalization were achieved. In addition, features such as early stopping, tree pruning, and column subsampling render the training process more stable and efficient. Owing to this robust structure, it was considered a high-performance classifier in our study.

**A light-gradient boosting machine (LightGBM)** is a boosting algorithm developed specifically to achieve high-speed training with lower memory usage, particularly for large datasets. One of the key features that plays an important role in the model’s performance is its use of a leaf-wise strategy instead of the traditional level-wise tree-growing strategy. This method provides faster convergence by expanding the branch, which reduces the largest loss at each step of the tree. In addition, owing to histogram-based decision splitting, both the processing time and memory consumption are significantly reduced. In this study, LightGBM was used to determine whether it provided an optimal balance between classification accuracy and training time.

**Categorical boosting (CatBoost)** was developed to provide high accuracy for datasets with categorical features. However, it also demonstrates highly effective performance for data structures with numerical features. A notable aspect of CatBoost is its use of the ordered boosting technique, which reduces variance depending on the order of training, and its symmetric tree structure, which helps to maintain the structural stability of the model. This approach enabled the generation of results that were resistant to overfitting. Additionally, the model structure, which exhibits low sensitivity to hyperparameter settings, offers ease of use in practice. In our study, the classification success of numerically derived feature matrices from images was experimentally tested.

The outputs of all training processes conducted with these models were analyzed using evaluation metrics such as accuracy, precision, recall, and F1-Score. By examining the obtained results, the best-performing feature extractor-classifier pairings were identified, and the significance levels were assessed using statistical tests.

In this study, to ensure a fair comparison and robust performance, each gradient boosting classifier was initialized with specific hyperparameters that are known to be effective for classification tasks. The models were not trained using their default library settings; instead, a carefully selected set of parameters was used to standardize the experimental setup and ensure its reproducibility. The final hyperparameter values used for each classifier are listed in Table 3.

**Table 3:** Summary of feature extraction configurations and classifier hyperparameters.

Feature Extractor	Extraction Layer	Feature Vector Size	Classifier	Tuned Hyperparameters
DenseNet121	Global Avg. Pool	1024	**XGBoost**	n_estimators: 200, lr: 0.1, max_depth: 5, subsample: 0.8
MobileNetV2	Global Avg. Pool	1280	**LightGBM**	n_estimators: 200, lr: 0.1, max_depth: −1, subsample: 0.8
EfficientNetV2B0	Global Avg. Pool	1280	**CatBoost**	iterations: 200, lr: 0.1, depth: 6
ConvNeXtTiny	Global Avg. Pool	768	**Grad. Boost**	n_estimators: 200, lr: 0.1, max_depth: 5, subsample: 1.0
			**AdaBoost**	n_estimators: 100, lr: 0.5

### Statistical tests

The success levels of the two models with the highest accuracy rates, obtained as a result of the combination of the feature extractor and classifier models, were statistically compared. The purpose of this test was to determine whether the observed performance difference was due to chance and to establish whether the superiority between the models was statistically significant. To determine whether the difference between the classification performances of the two feature extractor–classifier combinations is statistically significant, a comprehensive analysis was performed. Although k-fold cross-validation is widely used as a robustness measure in image classification studies, implementing it within the full experimental framework of this study (four feature extractors × five boosting algorithms × augmented and non-augmented settings) would require training hundreds of hybrid models, resulting in computational demands that contradict the primary objective of proposing a low-resource and accessible methodology. Therefore, instead of repeated retraining, we adopted a statistically rigorous and distribution-free bootstrap resampling strategy with 2,000 iterations, which provides stable performance estimates and has been recognized as a valid alternative for comparative model evaluation in machine learning research. This choice aligns with the study’s focus on computational efficiency while ensuring robust and reliable statistical validation of the proposed hybrid models. In this context, the bootstrap resampling method was applied to each model combination, and 2,000 accuracy values were generated for each. Using this method, statistical robustness was ensured for a reliable comparison of the accuracy performance distributions, without relying on parametric assumptions. In the first stage, the Shapiro-Wilk normality test was applied to examine the distribution characteristics of the obtained accuracy differences prior to the statistical test^42^. The test results indicated that the accuracy differences did not follow a normal distribution. Because this finding limits the validity of classic parametric tests, the analysis process continued with non-parametric tests to preserve statistical reliability. Accordingly, the Wilcoxon Signed-Rank test was applied to evaluate the difference between the accuracies of the two paired model groups^43^. This test was preferred because of its capacity to reliably evaluate even small differences when the distribution assumptions were not met. The statistical significance threshold was set at α = 0.05. The analysis revealed a statistically significant difference between the performances of the two models (*p* < 0.001), confirming the superiority of the proposed hybrid approach. This demonstrates that the hybrid model proposed in this study is superior from both observational and statistical perspectives.

This statistical validation process proves that the obtained classification performance is not the result of random chance but that the proposed model architecture genuinely offers a more effective and reliable solution. The detailed statistical findings, along with the comparison metrics are comprehensively presented in the Results section.

### Computational environment and implementation details

All experimental processes were performed on the cloud-based Google Colab platform, which offers researchers broad access^44^. Regarding the computational infrastructure, varying resource requirements were observed at different stages of the study. The training and testing of the baseline hybrid models (without data augmentation) were performed within the RAM capacities of standard Google Colab environments (approximately 12.3 GB). The data augmentation phase, which increased performance to 94%, required more memory (approximately 21.6 GB) due to the larger dataset, and a high-RAM cloud session was used for this stage. This two-stage approach demonstrates that the proposed model offers an accessible baseline solution for users with standard resources, while also being scalable to state-of-the-art performance with a moderate increase in computational capacity.

## Results

In this section, the results of the experimental studies conducted using the hybrid model approach are presented in detail. First, combinations of four different feature extraction deep learning models and five different machine learning classifiers before data augmentation, as listed in Table 4, were evaluated. In line with this structure, 20 different model combinations were created, and the classification performance of each was comparatively analyzed using various metrics (accuracy, sensitivity, specificity, F1-score, etc.)^45^.

**Table 4:** Performance results of the 20 model combinations formed by four feature extractors and five classifiers prior to data augmentation.

Feature Extractor	Classifier	Accuracy	Precision	Recall	F1-Score
MobileNetV2	AdaBoost	89%	90.33%	89%	89.33%
MobileNetV2	LightGBM	91%	90.33%	90.33%	90.33%
**MobileNetV2**	**XGBoost**	**91%**	**90.33%**	**90.66%**	**90.33%**
MobileNetV2	CatBoost	90%	90%	90.33%	90.33%
MobileNetV2	Gradient Boosting	90%	89.33%	89.66%	89.66%
DenseNet121	AdaBoost	91%	91%	90.33%	90.66%
DenseNet121	LightGBM	91%	91.66%	91.66%	91%
**DenseNet121**	**XGBoost**	**92%**	**92.66%**	**92%**	**92.33%**
DenseNet121	CatBoost	91%	91.33%	91%	91%
DenseNet121	Gradient Boosting	92%	92%	91.66%	91.66%
EfficientNetV2B0	AdaBoost	65%	68%	63.33%	62.66%
EfficientNetV2B0	LightGBM	72%	76.66%	71.33%	72%
EfficientNetV2B0	XGBoost	73%	77.33%	71.66%	72.33%
EfficientNetV2B0	CatBoost	69%	73.33%	68%	67.66%
EfficientNetV2B0	Gradient Boosting	73%	76.66%	72.33%	72.66%
ConvNextTiny	AdaBoost	64%	66%	62.33%	62%
ConvNextTiny	LightGBM	72%	73.66%	71%	71.33%
ConvNextTiny	XGBoost	72%	73.33%	71%	71.33%
ConvNextTiny	CatBoost	69%	70.66%	67%	67.66%
ConvNextTiny	Gradient Boosting	71%	72.66%	70.33%	70.66%

**Fig. 8:**
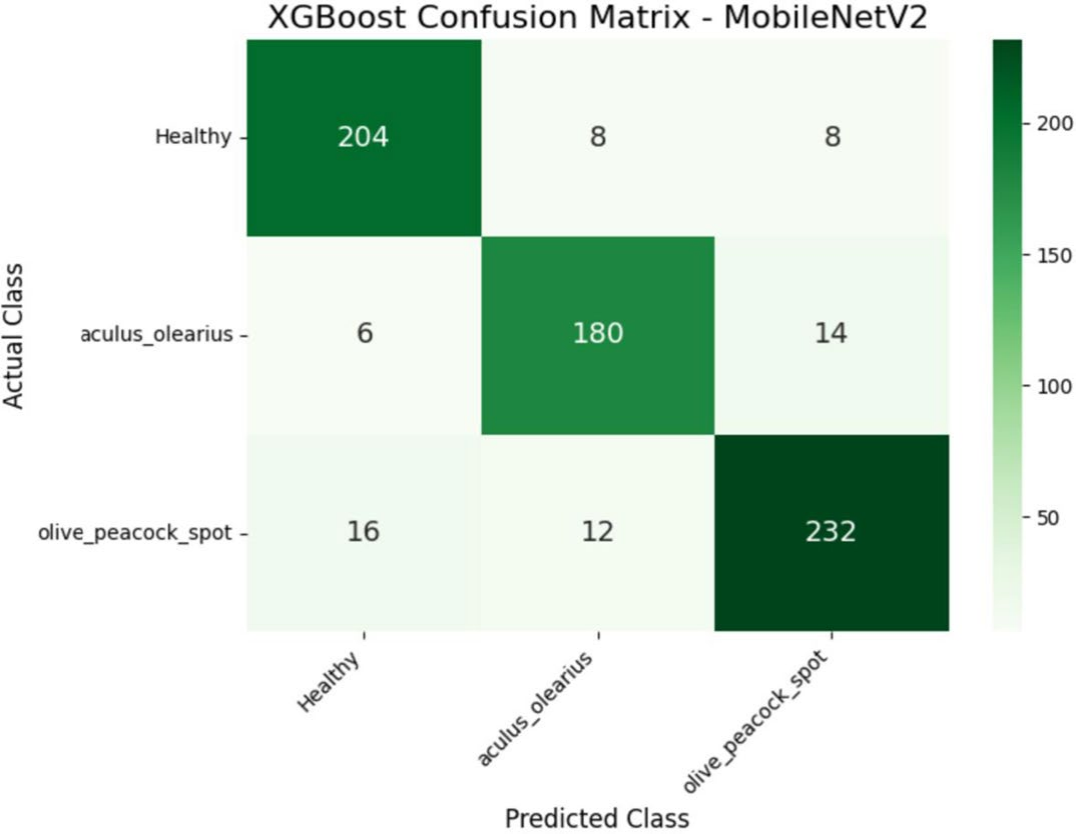
Confusion matrix of the MobileNetV2 + XGBoost hybrid model for class-level prediction performance.

**Fig. 9:**
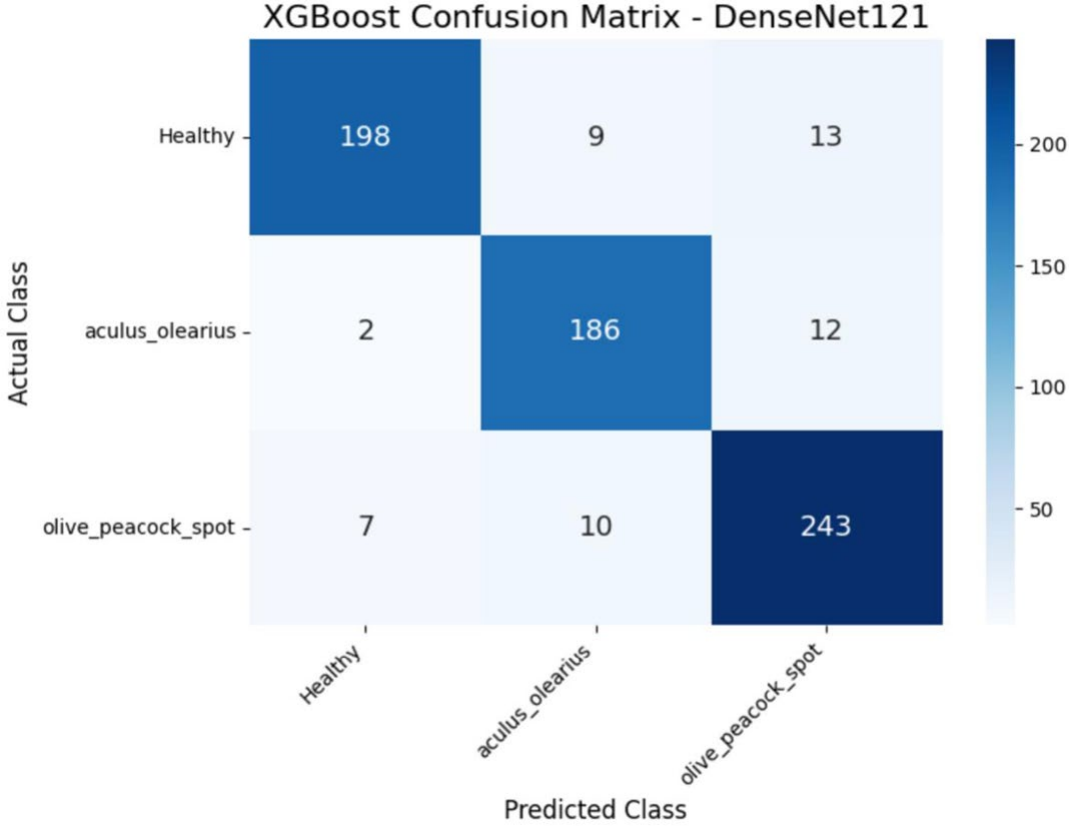
Confusion matrix of the DenseNet121 + XGBoost hybrid model for class-level prediction performance.

When the experimental findings were examined, it was observed that among the proposed hybrid structures, the model combinations MobileNetV2 + XGBoost and DenseNet121 + XGBoost exhibited the highest classification performances. These two structures stood out in terms of key performance metrics, such as accuracy, precision, recall, and F1-score, producing balanced results for all classes. The detailed class-level results obtained by these two models are presented in Tables 5 and 6, respectively. In particular, the DenseNet121 + XGBoost combination emerged as the most successful structure in this study, with an accuracy of 92% and an F1-score of 92.33%. To visualize the error patterns during the classification process, confusion matrices for both models are presented in Figs. 8 and 9.

**Table 5:** Class-based classification performance metrics for the MobileNetV2 + XGBoost hybrid model.

Class name	Precision	Recall	F1-Score	Support Count
Healthy	90%	93%	91%	220
Olive Peacock Spot	91%	89%	90%	260
Aculus Olearius	90%	90%	90%	200
Average/Total	90.33%	90.66%	90.33%	680

**Table 6:** Class-based classification performance metrics for the DenseNet121 + XGBoost hybrid model.

Class Name	Precision	Recall	F1-Score	Support Count
Healthy	96%	90%	93%	220
Olive Peacock Spot	91%	93%	92%	260
Aculus Olearius	91%	93%	92%	200
Average/Total	92.66%	92%	92.33%	680

### Impact of data augmentation

**Fig. 10:**
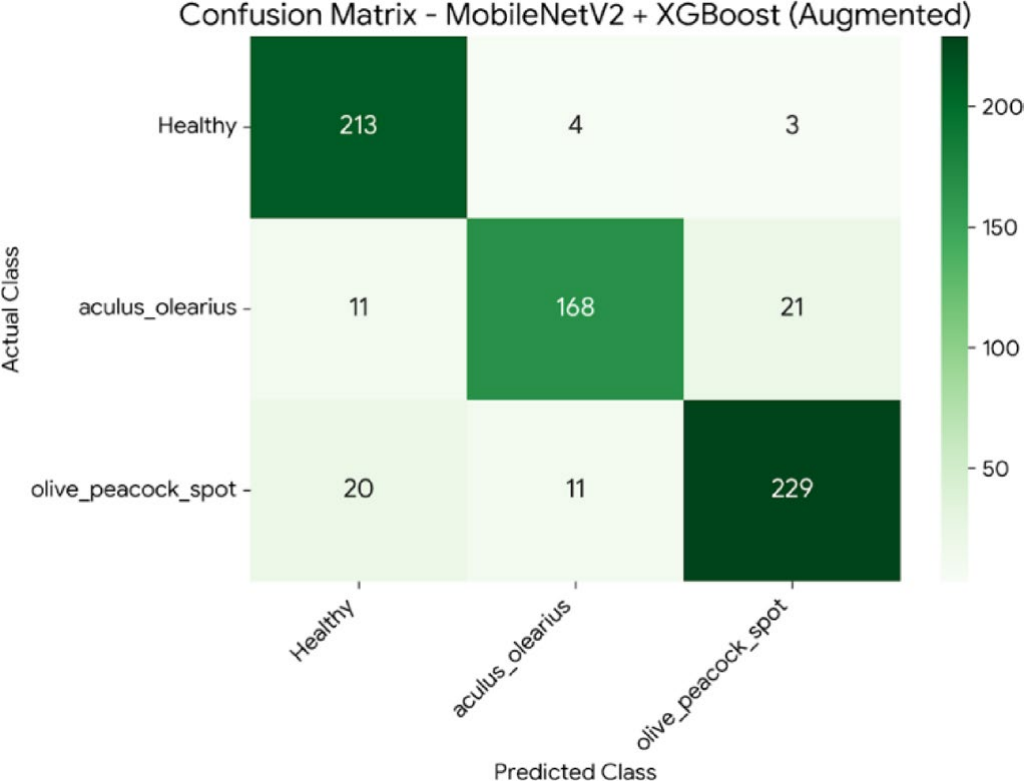
Confusion matrix for the MobileNetV2 + XGBoost hybrid model after training with data-augmented data.

**Fig. 11:**
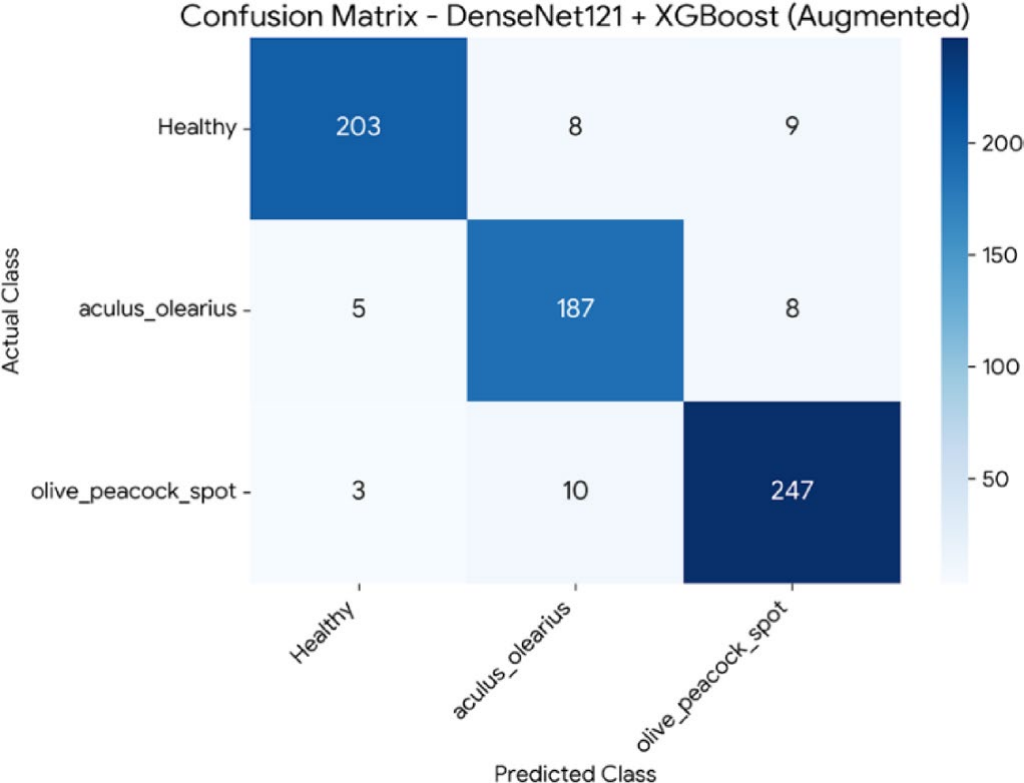
Confusion matrix for the DenseNet121 + XGBoost hybrid model, showing class-level prediction performance after training with data augmentation.

To investigate the potential for performance improvement and further test the robustness of the top-performing hybrid models, a data augmentation experiment was conducted. In this process, to synthetically double the size of the training set, a series of geometric transformations were applied using Keras’s ImageDataGenerator, including randomly rotating images by up to 20 °, randomly shifting them horizontally and vertically by up to 10%, applying shear and zoom transformations of up to 10%, and randomly flipping them horizontally. Any new pixels created were filled using the nearest available pixel value (fill_mode=’nearest’). Following this process, the two best models from the initial experiments, DenseNet121 + XGBoost and MobileNetV2 + XGBoost, were retrained on the augmented dataset and re-evaluated using the original test set. The comparative results of this experiment are presented in Table 7. The DenseNet121 + XGBoost model demonstrated a significant performance increase, with its accuracy increasing by 2% points to 94%. In contrast, the MobileNetV2 + XGBoost model showed a slight decrease in performance, suggesting that the effectiveness of augmentation is model-dependent. The confusion matrices detailing the class-level performance for these new results are presented for the MobileNetV2 + XGBoost model in Fig. 10 and for the DenseNet121 + XGBoost model in Fig. 11, respectively.

**Table 7:** Performance comparison before and after data augmentation.

Model	Metric	Score(Before Aug.)	Score (After Aug.)	Change
DenseNet121+ XGBoost	Accuracy	92%	94%	+2.00%
	F1-Score	92.33%	94%	+1.67%
MobileNetV2+ XGBoost	Accuracy	91%	89.71%	-1.29%
	F1-Score	90.33%	%90	-0.33%

### Statistical evaluation

In the initial evaluation of the classification results without data augmentation, the MobileNetV2 + XGBoost model achieved an accuracy of 91%. The DenseNet121 + XGBoost combination exhibited the highest performance, with an accuracy rate of 92%. The similar high performance exhibited by these two models necessitated the question of whether the difference between them was statistically significant. In this context, the statistical tests detailed in the Materials and Methods section were applied. The Wilcoxon Signed-Rank test revealed that the DenseNet121 + XGBoost combination was significantly superior to the MobileNetV2 + XGBoost combination (W = 100278.0, *p* < 0.001). To assess the practical significance of this difference, the effect size was measured using the Rank-Biserial Correlation (RBC), which was found to be RBC = −0.89. This value indicates a large effect size, confirming that the observed performance difference is not coincidental but rather a structural superiority arising from the architecture of the model.

Following data augmentation, a re-evaluation of the models confirmed the superior performance of the DenseNet121 + XGBoost combination, which achieved a new peak accuracy of 94%. In contrast, the MobileNetV2 + XGBoost model exhibited a decline in performance, with an accuracy of 89.71% on the augmented dataset. To ascertain whether the performance difference between the models remained significant after data augmentation, further statistical analysis was conducted. The Wilcoxon Signed-Rank test confirmed that the DenseNet121 + XGBoost combination significantly outperformed the MobileNetV2 + XGBoost combination (W = 4.5, *p* < 0.001). The practical significance of this gap was underscored by a Rank-Biserial Correlation (RBC) of −0.99, corresponding to a very large effect size. This outcome provides strong evidence that the observed performance difference is not due to chance but reflects a consistent structural advantage inherent in the DenseNet121-based architecture. Therefore, the DenseNet121 + XGBoost hybrid model was statistically validated as the most robust and reliable classification approach in this study, proving its efficacy on both the original and augmented datasets.

## Discussion

In this study, the hybrid model combining DenseNet121 and XGBoost confirmed its effectiveness by achieving a notable accuracy of 94% after data-augmentation. Although some state-of-the-art models in the literature report higher accuracy rates, the primary contribution of this study lies in demonstrating a strong balance between high performance and practical accessibility. This result, achieved without the need for specialized high-end hardware, positions the proposed method as a sustainable and scalable solution for real-world agricultural applications, aligning with the primary goal of this study of increasing technological accessibility for producers at various scales.

**Table 8:** Comparative analysis of the proposed hybrid model with state-of-the-art methods.

Study	Method/architecture	Reported accuracy	Key characteristics & requirements
Sarantakos et al. (2024)	Cloud Service (AWS Rekognition)	99.6%	Cloud-dependent; requires internet and has potential running costs.
Alshammari et al. (2023)	Hybrid (WOA + ANN)	98.9%	Requires complex optimization; potentially high computational cost.
Uğuz & Uysal (2021)	Custom CNN	95%	High performance is dependent on data augmentation.
Raouhi et al. (2022)	Hybrid (CNN + Optimization)	98.48%	Tested on a larger, more diverse dataset (6 disease types).
Our Approach	Hybrid (DenseNet121 + XGBoost)	94%	Flexible Approach: Baseline (92%) runs on standard instances (~ 12.3GB RAM); High-performance (94%) requires more memory (~ 21.6GB RAM).

Although the proposed hybrid model (DenseNet121 + XGBoost) achieves 94% accuracy, a figure numerically surpassed by some counterparts in the literature, it establishes a superior balance between practical applicability, computational efficiency and accessibility. Table 8 summarizes this comparative positioning. For instance, unlike the cloud-based service employed by Sarantakos et al., which reported 99.6% accuracy, our model obviates the need for constant Internet connectivity and potential running costs, which are critical barriers for in-field agricultural applications. Similarly, whereas the 98.9% accuracy of Alshammari et al. necessitates complex optimization and entails high computational costs, our approach circumvents these challenges. Notably, compared with the custom CNN architecture by Uğuz and Uysal (2021), which achieved 95% accuracy on the same dataset, our hybrid approach offers a significant architectural advantage: it decouples feature extraction (DenseNet121) from classification (XGBoost), thereby leveraging the power of transfer learning while mitigating the substantial computational burden and risk of overfitting associated with training a monolithic deep learning model from scratch. This design not only renders our solution accessible for operation on standard hardware but also makes it more scalable and sustainable for producers of various sizes, ultimately democratizing access to AI technology in agriculture.

All experimental processes were performed on the cloud-based Google Colab platform, which offers researchers broad and accessible computational resources. The study’s findings on resource consumption revealed a flexible trade-off between performance and computational requirements. The baseline model, which achieved 92% accuracy, required approximately 12.3 GB of RAM, confirming its feasibility for execution on standard, widely available cloud instances. To achieve a higher accuracy of 94%, the application of data augmentation naturally increased the memory footprint owing to the larger dataset, with a peak consumption of approximately 21.6 GB. Although this requirement exceeds that of standard free-tier environments, it remains well within the capabilities of readily available high-RAM cloud instances or modern workstations, thereby avoiding the need for specialized, high-end GPU hardware. This positions the proposed hybrid approach as a scalable solution, offering a highly accessible baseline for users with limited resources and a pathway to state-of-the-art performance for those with moderately increased computational capacities.

When examining the deep learning models used for feature extraction in this study, it was observed that the results were not directly related to the number of parameters in the model. For example, ConvNeXt-Tiny, which has one of the lowest classification performances, has approximately 28.6 million parameters and 50 layers, whereas the DenseNet121 model, which showed the highest success, operates with 8.1 million parameters but consists of a much deeper structure with 242 layers. Similarly, the second most successful model, MobileNetV2, consists of only 3.5 million parameters and 105 layers in total. In contrast, another model with low performance, EfficientNetV2B0, comprises 7.2 million parameters and approximately 55–60 layers. These findings suggest that model performance may be closely related not only to the parameter size but also to the architectural depth and layer connectivity. This underscores the need for a detailed examination of the factors affecting model performance, particularly on imbalanced datasets, and highlights the necessity for further research in this context.

The statistical superiority of the DenseNet121-based hybrid model can be attributed to its distinctive architectural design, which is characterized by dense connectivity. Unlike traditional architectures, DenseNet connects each layer to every other layer, thereby ensuring maximum information flow and facilitating feature reuse. This mechanism allows the model to preserve both low-level textural details, which are critical for identifying early stage disease spots, and high-level semantic information throughout the network. Furthermore, this finding highlights the critical role of parameter efficiency in raw model size. Larger models, such as ConvNeXt-Tiny (28.6 M parameters), despite their high capacity, face a higher risk of overfitting—memorizing noise rather than learning generalizable patterns—when trained on moderately sized agricultural datasets. In contrast, DenseNet121 achieved higher accuracy with significantly fewer parameters (8.1 M), suggesting that its compact architecture acts as a form of implicit regularization, ensuring that the learned features are robust and generalizable.

These results indicate that the developed hybrid model has the potential to detect diseases commonly found in olive groves, such as Olive Peacock Spot and Olive Bud Mite, before they spread. Early detection of these diseases not only limits their environmental impact by reducing pesticide use, but also increases productivity and supports economic sustainability by preventing crop loss. In this context, a roadmap is proposed for the integration of the model into the field: mobile application-based solutions should be developed for small producers, whereas integrated remote sensing systems with unmanned aerial vehicles are recommended for large producers.

### Computational efficiency analysis

To empirically validate the computational efficiency of the proposed framework, runtime benchmarks were performed in the Google Colab environment. The experimental results, as detailed in Table 9, demonstrate that the frozen-layer hybrid strategy significantly reduces the computational burden compared to traditional methods. The total training time for the DenseNet121 + XGBoost model (including feature extraction on augmented data) was approximately 2–3 min. In contrast, full fine-tuning of deep architectures typically requires several hours because of the high cost of backpropagation. Furthermore, the peak GPU memory (VRAM) usage was observed to be approximately 8 GB, confirming that the model can be trained on standard, free-tier cloud resources or mid-range consumer hardware, fulfilling the study’s objective of accessibility.

**Table 9:** Computational efficiency metrics of the proposed DenseNet121 + XGBoost model compared with traditional fine-tuning benchmarks.

Metric	Proposed hybrid model	Traditional full fine-tuning (estimated)*
Total Training Time	2–3 min (Feature Extraction + Boosting)	3–4 h (Full Backpropagation)
Inference Time	9–10 ms/image	9–10 ms/image
GPU VRAM Usage	8 GB (Compatible with Standard T4/RTX Cards)	> 24 GB (Often Requires High-End GPUs)
Computational Load	Low (Frozen Backbone, No Backprop)	Very High (Gradient Intensive)

## Conclusions

This study successfully addressed the challenge of high computational costs in deep learning for agricultural applications by developing and validating an effective and low-cost hybrid model. The experimental findings confirmed that the combination of a DenseNet121 feature extractor with an XGBoost classifier yielded the most robust and accurate results. The model established a strong baseline accuracy of 92%, which was further enhanced to 94% through data augmentation, demonstrating a clear pathway for performance optimization. Key insights from this study include the model-dependent nature of data augmentation and the confirmation that architectural efficiency can be more critical than the raw parameter count, reinforcing the superiority of DenseNet121 for this task.

Ultimately, this study presents more than just a classification tool; it offers a flexible framework that can be adapted to different operational constraints. It provides a highly efficient solution for users with standard resources and a method to achieve state-of-the-art performance for those with access to moderate memory sizes. This scalability is a significant contribution towards democratizing AI in agriculture, supporting sustainable practices by enabling early disease detection, and enhancing the economic viability of small- and medium-sized producers. In future studies, we plan to adapt this successful framework to different agricultural products and larger datasets, systematically examine the effects of various data augmentation techniques, and implement it with field-specific mobile or drone-based applications.

## Data Availability

The data supporting the findings of this study are publicly available from the Olive Leaf Image Dataset: [https://www.kaggle.com/datasets/habibulbasher01644/olive-leaf-image-dataset](accessed on 2 May 2025).

## Code availability

The custom code used for data preprocessing, feature extraction, model training and evaluation in this study is not publicly available due to institutional and confidentiality constraints. Nevertheless, it can be provided by the corresponding author upon reasonable request for academic and non-commercial research purposes.
